# Visible Light-Generated Antiviral Effect on Plasmonic Ag-TiO_2_-Based Reactive Nanocomposite Thin Film

**DOI:** 10.3389/fbioe.2021.709462

**Published:** 2021-09-29

**Authors:** Zsolt Boldogkői, Zsolt Csabai, Dóra Tombácz, László Janovák, Lilla Balassa, Ágota Deák, Péter S. Tóth, Csaba Janáky, Ernő Duda, Imre Dékány

**Affiliations:** ^1^ Department of Medical Biology, Faculty of Medicine, University of Szeged, Szeged, Hungary; ^2^ Department of Physical Chemistry and Materials Science, University of Szeged, Szeged, Hungary; ^3^ Department of Physical Chemistry and Materials Science, Interdisciplinary Excellence Centre, University of Szeged, Szeged, Hungary

**Keywords:** photoreactive composite film, photooxidation, herpesvirus, pseudorabies virus, antiviral surface, epidemic prevention and control

## Abstract

The recent coronavirus pandemic pointed out the vulnerability of humanity to new emerging infectious diseases. Experts warn that future pandemics may emerge more frequently with greater devastating effects on population health and the world economy. Although viruses are unable to propagate on lifeless surfaces, they can retain their infectivity and spread further on contact with these surfaces. The objective of our study is to analyze photoreactive composite films that exert antiviral effects upon illumination. Reactive plasmonic titanium dioxide-based polymeric nanocomposite film was prepared with a thickness of 1–1.5 µm, which produces reactive oxygen species (ROS) under visible light irradiation (*λ* ≥ 435 nm). These species are suitable for photooxidation of adsorbed organic molecules (e.g., benzoic acid) on the nanocomposite surface. Moreover, high molecular weight proteins are also degraded or partially oxidized in this process on the composite surface. Since the Ag^0^-TiO_2_/polymer composite film used showed excellent reactivity in the formation of OH• radicals, the photocatalytic effect on high molecular weight (M = ∼66.000 Da) bovine serum albumin (BSA) protein was investigated. Given that changes in the structure of the protein were observed upon exposure to light, we assumed virucidal effect of the illuminated photoreactive composite film. We tested this hypothesis using an airborne-transmitted herpesvirus. As a result, we obtained a drastic decrease in infection capability of the virus on the photoreactive surface compared to the control surface.

## Introduction

Antimicrobial nanomaterials have achieved a great scientific interest world-wide (Rodríguez-Baño et al., 2013; 2. Grau et al., 2013). The increasing application of nanoparticles as antimicrobials has been reviewed in industries, medicine, cosmetics, textiles and food packaging (Gajjar et al., 2009; Chorianopoulos et al., 2011). Titanium dioxide-based nanoparticles are well known that under UV light irradiation produce free radicals, which cause decomposition of organic molecules through oxidation (Fujishima and Honda, 1972; Paul et al., 2007). Titanium dioxide has been developed as a photocatalyst and proved to be its antibacterial effect under UV light irradiation (Chih-Yu et al., 2010; Veres et al., 2012a). The antibacterial activity of TiO_2_ is due to the photocatalytic generation of strong oxidizing power when illuminated with UV light (Chorianopoulos et al., 2011). Surfaces with TiO_2_ or other photocatalyts content is able to kill prokaryotic organisms due to their photocatalytic properties (Györgyey et al., 2016). However, it has also been reported that due to its wide bandgap (>3.2 eV), titanium dioxide is only excited by UV light (*λ* < 380 nm), which amounts to about 5% of natural sunlight (Khore, et al., 2018). It is possible to extend the absorption spectrum of TiO_2_ by, for example, modifying or doping the catalyst with different plasmonic metals (Veres et al., 2012b). We have reported several times, that the functionalization of initial semiconductor photocatalyst particles (e.g., TiO_2_ or ZnO) with plasmonic (e.g., Au or Ag) nanoparticles provides samples with improved photocatalytic properties under visible light irradiation (Kőrősi et al., 2008; Veres et al., 2012a; Veres et al., 2012b).

During the photocatalytic process, the irradiated photocatalyst particles produce highly reactive oxygen species such as superoxide radical ion (O_2_•^−^), hydrogen peroxide (H_2_O_2_), or hydroxyl radical (HO•) (Martra et al., 1999). Due to these light induced formation of reactive radicals, the irradiated photocatalyst particles can degrade many organic compounds (Kun et al., 2009; Samu et al., 2017) and inactivate bacterial cells via destroying the cell wall and their DNA (Tallósy et al., 2014a). Hence, titanium dioxide- based plasmonic photocatalysts have become one of the most intensively studied photocatalysts in the past decades (Gupta et al., 2012).

As a prelude to this study, first we optimized the photocatalyst composition (silver or gold content on the TiO_2_ or ZnO particles) of the plasmonic photocatalyst (Kőrősi et al., 2008; Veres et al., 2012a; Veres et al., 2012b; Veres et al., 2014). Next, we also tested a lot of polymer for the photocatalyst immobilization such us poly-(2-hydroxyethyl-acrylate) (Mérai et al., 2018), poly(ethyl acrylate-co-methyl methacrylate) (Veres et al., 2014), or poly-(1H,1H,2H,2H-perfluorodecyl-acrylate) (Mérai et al., 2019). At the end of this systematic exploration it has been established that the appropriate coating material presented here contains Ag^0^-TiO_2_ plasmonic photocatalyst with 0,5 wt% Ag content as active agent, while the photocatalyst immobilization can be achieved by poly(ethyl acrylate-co-methyl methacrylate) polymer and the optimal photocatalyst/polymer ratio is 60/40 wt% in the hybrid layer. This film showed obvious antibacterial properties against *Staphylococcus aureus*, Enterococcus faecium, *Pseudomonas aeruginosa*, *Acinetobacter* baumannii, or Methicillin-resistant *Staphylococcus aureus* (MRSA) (Tallósy et al., 2014a; Tallósy et al., 2014b; Tallósy et al., 2016). Thus, it also seems reasonable to study the antiviral effect of the layer.

Based on the above results, it is intriguing to investigate the antiviral effects of the photoreactive surfaces, as the alteration of the protein structure caused by the reactive radicals produced under the photocatalytic process provides an opportunity to reduce or eliminate the infectivity of the virus. Although the antiviral properties of the light- induced TiO_2_ and even plasmonic Ag^0^-TiO_2_ photocatalysts has already been reported (Akhtar et al., 2019; Moongraksathum et al., 2019), here we demonstrate that it is also possible to immobilize the photocatalyst particles with a suitable polymeric binder material and the obtained composite layer shows also obvious antiviral behavior. In addition, the improved mechanical properties and enhanced durability of the composite layer ensured by the polymer also enables the potential practical use of the coating. In this study, we applied pseudorabies virus (PRV; Tombácz et al., 2018), an alphaherpesvirus, closely related to the human pathogen; varicella-zoster virus (Prazsák et al., 2018), and herpes simplex virus (Tombácz et al., 2020). They infect different hosts, however are remarkably similar in structure, replicative cycle, and ingress into cultured cells. Herpesviruses are enveloped viruses containing large DNA genomes. These viruses spread mainly through a direct inhalation of droplets, and indirectly from contacting infected surfaces followed by touching the nose or the lips. In this report, PRV was used as a model virus for the investigation of the effect of the photocatalytic surface on the infectivity of the virus.

## Materials and Methods

### Preparation of Photoreactive Hybrid Thin Film

The silver-modified photocatalyst was prepared by directly functionalizing TiO_2_ (Aeroxide P25” CAS No. 13463-67-7) with 0.5 wt% AgNP (Veres et al., 2012). Poly(ethyl acrylate-co-methyl methacrylate) (p(EA-co-MMA) polymer was used for the preparation of photocatalyst/polymer nanohybrid films (Tallósy et al., 2014a). During the composite film synthesis, 10 wt% aqueous suspensions of Ag^0^-TiO_2_ photocatalyst and polyacrylate binder were prepared and evenly sprayed on glass substrates and on microtiter plates from a distance of 15 cm. A gravity feed airbrush (ChroMax BD-203, United Kingdom) was used to prepare the photoreactive composite coatings. This airbrush allows the suspension to be drawn down into the body of the airbrush by gravity, where it then mixes with the high pressure of air sprayed onto the holder. For photocatalytic (5 cm^2^ × 5 cm^2^) and microbiological measurements the nanohybrid films (1 mg/cm^2^) were fabricated on glass and on microtiter plates. The mass ratio of photocatalyst particles/polymer binder was 60:40 wt% in each case. Pure Ag-TiO_2_ photocatalyst and pure polyacrylate thin films with the same specific mass (1 mg/cm^2^) were also prepared for control photocatalytic and antiviral measurements, respectively.

## Methods of Characterization

The synthesized Ag^0^-TiO_2_ nanoparticles were studied using FEI Tecnai G2 20 X-Twin (United States) type transmission electron microscope (TEM), operating at 200 kV acceleration voltage. The 0.01% aqueous sample was dropped and dried on a copper-mounted carbon film (with 200 mesh, lacy carbon film 200 Mesh Cu) for TEM measurements. The structure of the Ag-TiO_2_ and AgTiO_2_/polymer thin films were examined by scanning electron microscope (SEM, Hitachi S-4700 microscope, Japan), applying a secondary electron detector and 10 kV acceleration voltage. Energy dispersive X-ray spectra were measured using the Röntec EDX detector at 15 keV.

The optical characterization of the TiO_2_ and Ag^0^-TiO_2_ powders, diffuse reflectance UV-Vis spectra were recorded with a CHEM 2000 UV–Vis (Ocean Optics Inc., United States) spectrophotometer equipped with an integrated sphere. The layer thickness values of the prepared thin films were measured with an Elcometer 224 type (Germany) digital profile gauge. The light intensity on the irradiated surface of the photoreactive nanohybrid films was measured with a power meter (Thorlabs GmbH, Germany). During the measurement the distance of the light source from the surface was systematically increased and measured the corresponding light intensity values. Thus, we determined how light intensity changes with increasing distance from the light source.

During the photoelectrochemical activity (PEC) measurements the bare TiO_2_ and Ag^0^-TiO_2_ photocatalyst dispersions were spray-coated on ultrasonically cleaned (5–5 min in acetone (CAS No. 67-64-1) and isopropanol (CAS No. 67-63-0)) fluorine doped tin oxide (FTO) coated glass electrodes (Sigma-Aldrich, surface resistivity ∼7 Ω/sq). The FTO electrodes were masked to have an exposed surface area of 1 cm^2^ during the spray-coating process as the electrochemically active area. All PEC measurements were carried out with a Biologic VMP-300 potentiostat/galvanostat (BioLogic, France) in a typical three-electrode setup. The TiO_2_ and Ag^0^-TiO_2_ modified FTO electrodes were used as working electrodes for the PEC measurements, while a Pt plate, and Ag/AgCl/3 M NaCl were applied as counter, and reference electrodes, respectively. A Newport LCS-100 type solar simulator (G2V Optics, Canada), and a Hamamatsu LC-4 type 300 W Hg −Xe arc-lamp (Hamamatsu Inc., Japan) were used as the light sources. The radiation source was placed 18 cm (solar simulator) and 13 cm (Hg–Xe arc-lamp) away from the illuminated working electrode surface ensuring the 100 mW/cm^2^ flux. The cell contained aqueous solution of 0.1 M Na_2_SO_4_ (CAS No. 7757-82-6) and 5v/v% methanol (CAS No. 67-56-1), and was saturated with Ar for 30 min before each measurements. Photovoltammograms were recorded using 5 mV/s potential sweep in parallel with periodically interrupted irradiation (0.1 Hz).

**FIGURE 1:**
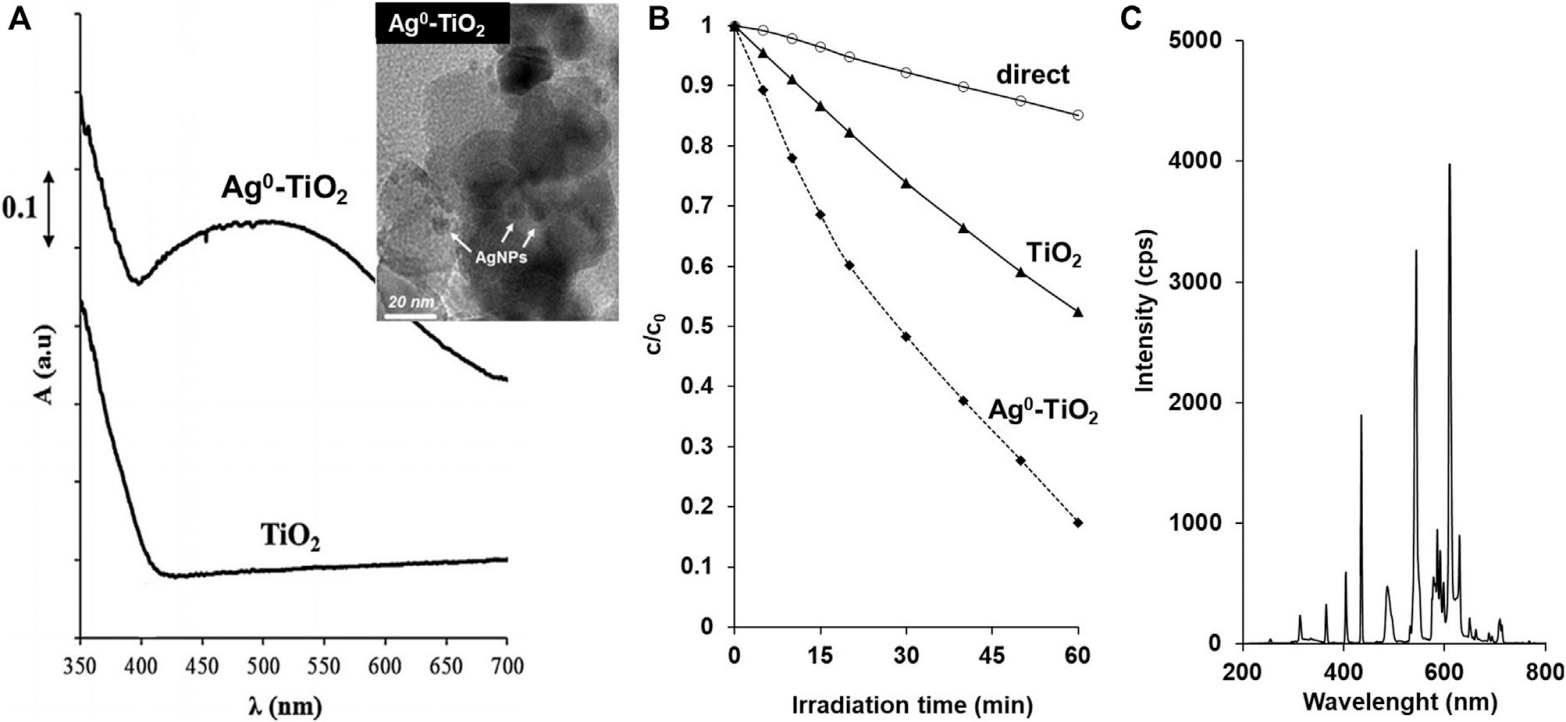
The diffuse reflectance spectra of the initial P25 TiO_2_ and plasmonic Ag^0^-TiO_2_ photocatalyst (0.5 m/m% surface Ag^0^ content) with the TEM picture of Ag^0^-TiO_2_ photocatalyst **(A)**, photocatalytic degradation of ethanol vapour on pure photocatalyst films **(B)** as well as the spectral distribution of the light source used in this experiment (LightTech) **(C)**.

The photocatalytic activities of the prepared pure Aeroxide P25 TiO_2_ and the plasmonic Ag^0^-TiO_2_ layers were verified with ethanol (Merck, CAS No. 64-17-5) (as test molecules) degradation tests under visible light illumination (LightTech light source, Hungary) (Figure 1C) at the solid/gas interface. Photooxidation of ethanol vapour on catalyst films was performed in a circulation reactor (volume c. a. 165 ml) at 25.0 ± 0.1°C. The light source was fixed at 50 mm distance from the films. After injection of ethanol and water vapour, the system was left to stand 30 min for the establishment of adsorption equilibrium on the surface of films. The composition of vapour phase was analysed by gas chromatograph (Shimadzu GC-14B, Japan) equipped with a thermal conductivity (TCD) and a flame ionisation detector (FID). The initial concentration of ethanol was 0.36 ± 0.018 mmol L^−1^ at relative humidity of ∼70%. During the measurements, the c/c_0_ values were determined as a function of illumination time, where c is the concentration of ethanol at time (t) and c_0_ is the initial concentration (c_0_ = 0.36 ± 0.018 mmol L^−1^).

The amount of hydroxyl radicals was measured from the reaction of luminol and hydrogen peroxide. The results were calculated from the chemiluminescence (CL) data with Sirius L Single Tube luminometer (Berthold Detection Systems, Hungary). Six milligrams of luminol (Sigma-Aldrich. CAS No. 521-31-3) was diluted in 1 ml of sodium hydroxide (0.1 M) (CAS No. 1310-73-2) and filled out to 20 ml with distilled water. The nanohybrid films were immersed in 40 ml of distilled water, then illuminated and shaken continuously during the experiment using a magnetic stirrer. Samples were taken after 60 min of illumination, 100 μL of the samples was added to 100 μL of luminol solution, and the intensity of the chemiluminescence was measured immediately with the luminometer (Hirakawa and Nosaka, 2002; Tallósy et al., 2014a). Based on the previously determined calibration curve (0–5 mM), the concentration of OH radicals is directly proportional to the measured RLU values as follows:

$$CH_{2}O_{2}\left(mM\right)=\text{measured}\;\text{RLU}\;\text{value}/41866,R^{2}=0.9977.$$ (1)

For quantitative characterization of the free radical concentration from the RLU data, the calculated equivalent concentration of H_2_O_2_ (mM) (CAS No. 7722-84-1) is displayed as a function of illumination time with the used light source (15 W low pressure mercury lamp (LightTech, Hungary) with characteristic emission wavelength at λ_max_ = 435 nm, Figure 1C) at 25.0 ± 0.5°C. The distance of the light source from the nanohybrid films was systematically changed in order to determine how the surface reactive oxygen species concentration changes with increasing distance from the light source.

The photocatalytic activities of the prepared Ag^0^-TiO_2_/polymer film was verified with benzoic acid (CAS No. 65-85-0) (as test molecules) and bovine serum albumin (BSA, as test protein, CAS No. 9048-46-8) degradation tests under visible light illumination (LightTech light source) at the solid/liquid interface. BSA was purchased from Sigma-Aldrich fraction V lyophilized powder, ≥98% (agarose gel electrophoresis), essentially fatty acid-free and essentially globulin-free. During the measurements, the microtiter plates were coated with Ag^0^-TiO_2_/polymer film (where the mass ratio of photocatalyst/polymer was 60:40 wt%) and 2 ml of 20 ppm benzoic acid and 100 ppm of BSA aqueous solution were placed in each cylindrical sample holder under continuous shaking. After 15 min adsorption time in dark, the microtiter plate with hybrid coating was irradiated with visible light (LightTech light source). The photocatalytic degradation of benzoic acid and BSA were recorded with a diode array spectrophotometer (Ocean Optics USB 2000; United States) in a 1 cm quartz cuvette. The fluorescence spectra of the degradation of BSA were recorded by a Horiba Jobin Yvon Fluoromax-4 spectrofluorometer (Japan) (excitation at 280 nm) in the 300–550 nm range using a 1 cm quartz cuvette. The photodegraded benzoic acid and BSA concentrations were quantified by the previously determined spectrophotometric calibration curve at a wavelength maximum of λ_benzoic ac_. = 273 nm and λ_BSA_ = 350 nm. During the measurements, the c/c_0_ values were determined as the function of illumination time, where c is the concentration of benzoic acid and BSA at time (t) and c_0_ is the initial concentration (in the case of benzoic acid the c_0_ = 20 ppm and in the case of BSA the c_0_ = 100 ppm).

For monitoring the conformational changes in the secondary structure of proteins, Circular Dichroism (CD) spectra were recorded (190–260 nm; 25.0°C; bandwidth: 2 nm; scanning speed: 100 nm/min) using a Jasco J-815 CD spectrometer (Japan).

To evaluate the abrasion resistance of coatings the taber abraser test is frequently used (Rossi et al., 2009). The abrasion tests were carried out with a 418 type manual Taber Abraser (United States). During the measurement the Ag-TiO_2_ and Ag-TiO_2_/polyacrylate (=60:40 wt%) photocatalyt layer with 1 mg/cm^2^ specific surface mass was abraded and the percentage weight loss of the tested surfaces were measured as a function of abrasion cycle.

### Cells and Viruses

An immortalized porcine kidney epithelial cell line (PK-15; ATCC^®^ CCL-33™) was used for the propagation of strain Kaplan of pseudorabies virus (PRV-Ka). Cells were cultivated in DMEM (Dulbecco’s Modified Eagle Medium; Gibco/Thermo Fisher Scientific) supplemented with 5% fetal bovine serum (Gibco/Thermo Fisher Scientific, CAS No. 9048-46-8) and 80 μg of gentamycin per ml (Gibco/Thermo Fisher Scientific, CAS No. 1403-66-3) at 37°C in the presence of 5% CO_2_. The virus stock was prepared by infecting PK-15 cells with the virus using 0.1 multiplicity of infection [MOI = plaque-forming units (pfu)/cell]. Viral propagation was allowed until complete cytopathic effect was observed. It was followed by three successive cycles of freezing and thawing of infected cells for releasing the viruses from the cells.

### Experimental Design

In total, 100 μL of the virus inoculum were sprayed onto the plates with photoreactive or control surfaces using a simple device, which produced aerosol and droplet particles with varying sizes, which mimics the natural spreading conditions of airborne viruses. The plates were either illuminated or kept in dark during the experiment. The light spectrum used in this experiment is illustrated at Figure 1C. The experiment was carried out at two temperatures [room temperature (RT) or 4°C] and at two incubation periods (before desiccation and after it) in both the photoreactive and control surfaces. Every experiment was carried out in three biological replicates. The light source was spaced 15 cm apart of the surfaces.

### Determination of the Virus Infectivity

After the incubation, the viruses were collected from the plates by pipetting 1,000 μL DMEM solution onto the infected area of the surface. The tissue Culture Infectious Dose (TCID50) values were determined in six parallel experiments using 96 microtiter plates and using the “Reed and Muench” and “Spearman-Karber” log10 50% end point dilution method (Ramakrishan, 2016):

Log1050% end point dilution=[(total number of positive wells/number of wells inoculated per dilution)+0.5]×log dilution factor. (2)

## Results

### Formation of Free Radicals on Photoreactive Hybrid Thin Film

**FIGURE 2:**
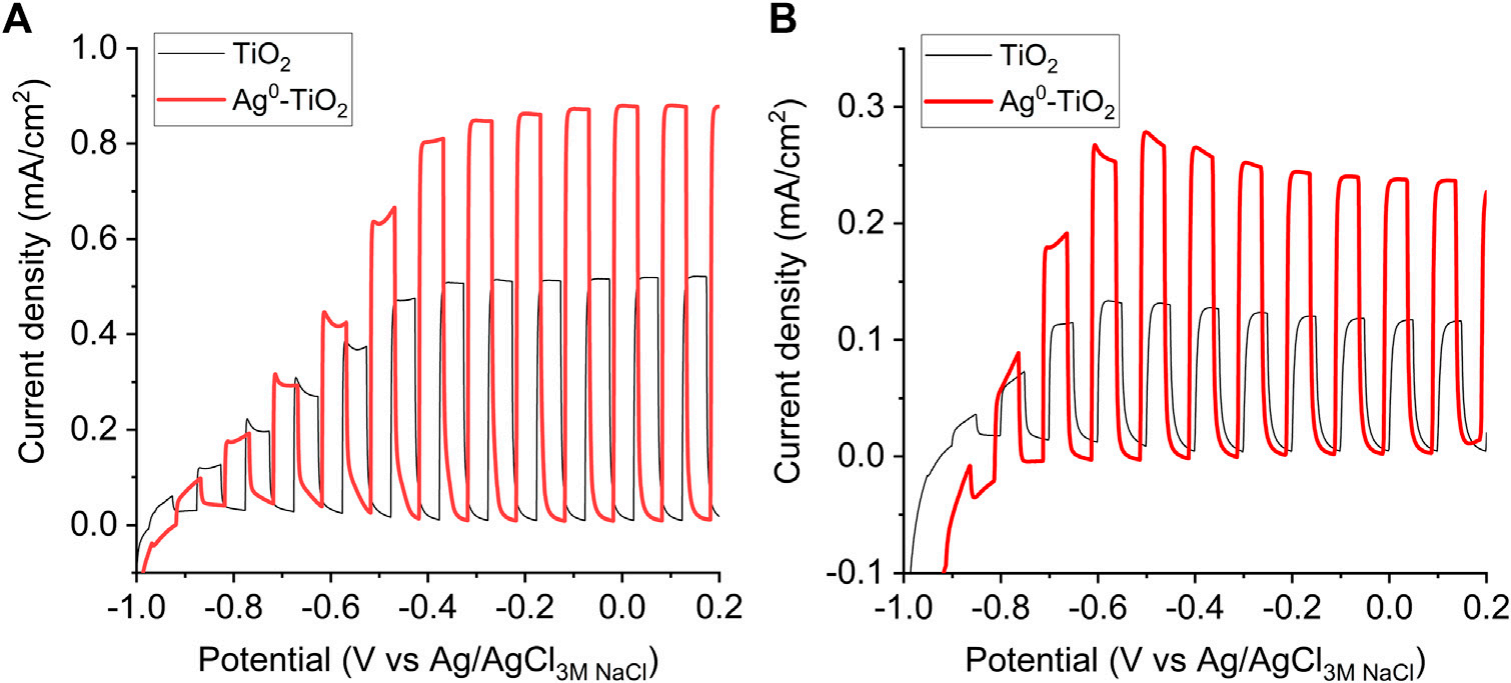
Photoelectrochemical activity of TiO_2_ and Ag^0^-TiO_2_ films in 0.1 M Na_2_SO_4_ with 5 v/v% methanol aqueous solution. Photovoltammograms recorded using solar simulator **(A)** and Hg −Xe arc-lamp **(B)** irradiation at a scan rate of 5 mV/s.

It has been reported that the plasmonic photocatalysts shows enhanced photocatalytic activity under visible light irradiation (Veres et al., 2012b; Tallósy et al., 2014a; Tallósy et al., 2016). This is due to the appearing plasmonic peak at λ = 450 nm (Figure 1A) and the resulted lower band gap energy (3.2 eV for initial TiO_2_ and 3.12 eV for plasmonic Ag^0^-TiO_2_ photocatalyst). As a results, the Ag^0^-TiO_2_ doped Aeroxide P25 semiconductor showed significantly higher rate of photooxidation for ethanol test molecule than pure TiO_2_ photocatalyst (Figure 1B) as reported several times in our previous papers (Veres et al., 2014; Veres et al., 2012a; Veres et al., 2012b; Kőrösi et al., 2008; Tallósy et al., 2014a). It is also worth to note that the oxidation state of the AgNPs on TiO_2_ could be change during the irradiation, however, this has no effect on the photocatalytic properties, because we also reported that plasmonic Ag-TiO_2_ semiconductors with different oxidation state (Ag^0^, AgO, Ag_2_O) of the AgNPs showed similar reaction rates for the photodegradation of ethanol under the same experimental condition (Veres et al., 2014). Thus, that the functionalization of the initial ∼20–40 nm TiO_2_ photocatalyst particles with ∼5–10 nm surface silver nanoparticles (AgNPs) resulted in a plasmonic photocatalyst, which shows enhanced photocatalytic activity under visible light irradiation (Veres et al., 2012b). The presence of silver and silver oxide on the surface of titania was also proved by XPS measurements (Veres et al., 2014; Kőrösi et al., 2008). According to the XPS spectra Ag 3d_5/2_ and Ag 3d_3/2_ components at 367.9 and at 373.9 eV, respectively, suggested the presence of silver oxide (Ag_2_O) on the surface of TiO_2_ with 87% anatase/13% rutile contents (Kőrösi et al., 2008). Moreover, the presence of AgNPs on the surface of titania was influenced the PEC properties of the samples, as well. Figure 2. shows the comparison of the PEC performances of TiO_2_ and Ag^0^-TiO_2_ films oxidizing 5 v/v% methanol in 0.1 M Na_2_SO_4_ solution under chopped light illumination (both with a solar simulator (Figure 2A) and a Hg−Xe arc-lamp (Figure 2B). The methanol was used as an organic hole scavenger to mimic the organic substance (virus genome) oxidation in aqueous medium in the other experiments. The shape of the photovoltammograms and the similar onset potentials (where the photocurrent develops) suggest the similar band structures and elementary processes: 1) absorption of a photon and generation of an electron-hole pair via the illumination, and 2) hole-transfer from the valence band of TiO_2_ to the electrolyte to oxidize methanol (Jeong et al., 2021). Under solar irradiation (Figure 2A), the photocurrents (which translates to the reaction rate in these processes) were ca. 0.13 mA/cm^2^ and 0.28 mA/cm^2^ at −0.5 V (vs. Ag/AgCl) in the case of TiO_2_ and Ag^0^-TiO_2_ electrodes, respectively (this is a 110% increase for the Ag-decorated electrodes). Under UV-light (Figure 2B), higher photocurrents were detected, but the relative increase was lower (∼67%). This higher PEC activity for the Ag^0^-TiO_2_ versus to bare TiO_2_ can be explained by at least two factors: 1) sensitizing effect of the plasmonic Ag nanoparticles (see the larger relative increase under sunlight), and 2) the suppression of electron-hole recombination. Since the photoreactive surfaces produced by us can be also activated by visible light excitation and photoreactive species are formed as a result of this process, a visible light source was employed for the experiments (Figure 1C). The spectrum shows that high light intensity characteristic peaks are in the wave-length range of visible light.

**FIGURE 3:**
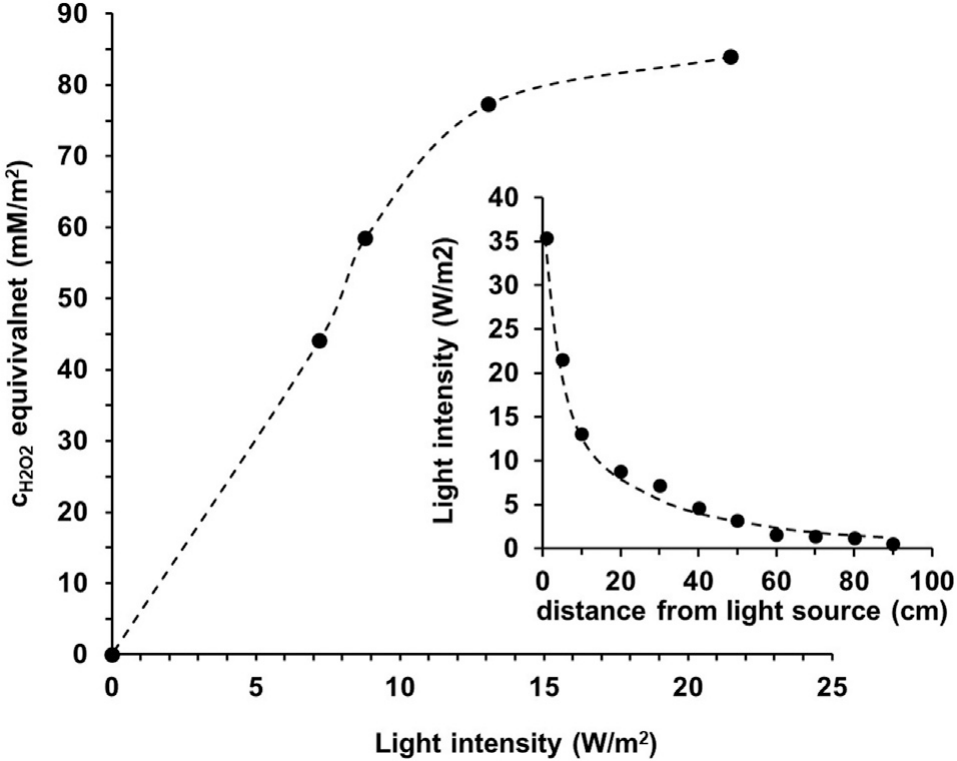
The effect of light intensity on the surface ROS concentration produced under irradiance of the photoreactive layer. The inserted figure shows the distance dependence of light intensity (here the dashed line is guide to eyes).

Considering the potential antiviral application of the photoreactive coating, it is also an important question how many radicals are formed on the surface depending on the light intensity. In order to answer this question we determined the light intensity values as a function of irradiation distance from the used light source. As it can be seen the measured light intensity is inversely proportional to the square of the distance from the source (Figure 3, inserted graph.). In parallel, the reactive oxygen species (ROS) concentration values were also measured at given distances via luminometric measurements (Tallósy et al., 2014a). Figure 3 shows that the measured ROS concentration values (expressed as H_2_O_2_ equivalent) increases almost linearly up to ∼13 W/m^2^ light intensity, then a constant value (∼80 mM/m^2^ H_2_O_2_ equivalent) is taken. For comparison, the average solar irradiance value is about 1000 W/m^2^, however,—according to our measurement—the light intensity values experienced in indoor environment (∼5–40 W/m^2^; Apostolou et al., 2016) are also sufficient for the generation of reactive species on the photocatalytic coating material. Thus, it can be concluded that even at relatively low light intensities, a sufficient amount of radicals is formed on the irradiated photoreactive surface.

### Photocatalytic Property of the Hybrid Thin Film

**FIGURE 4:**
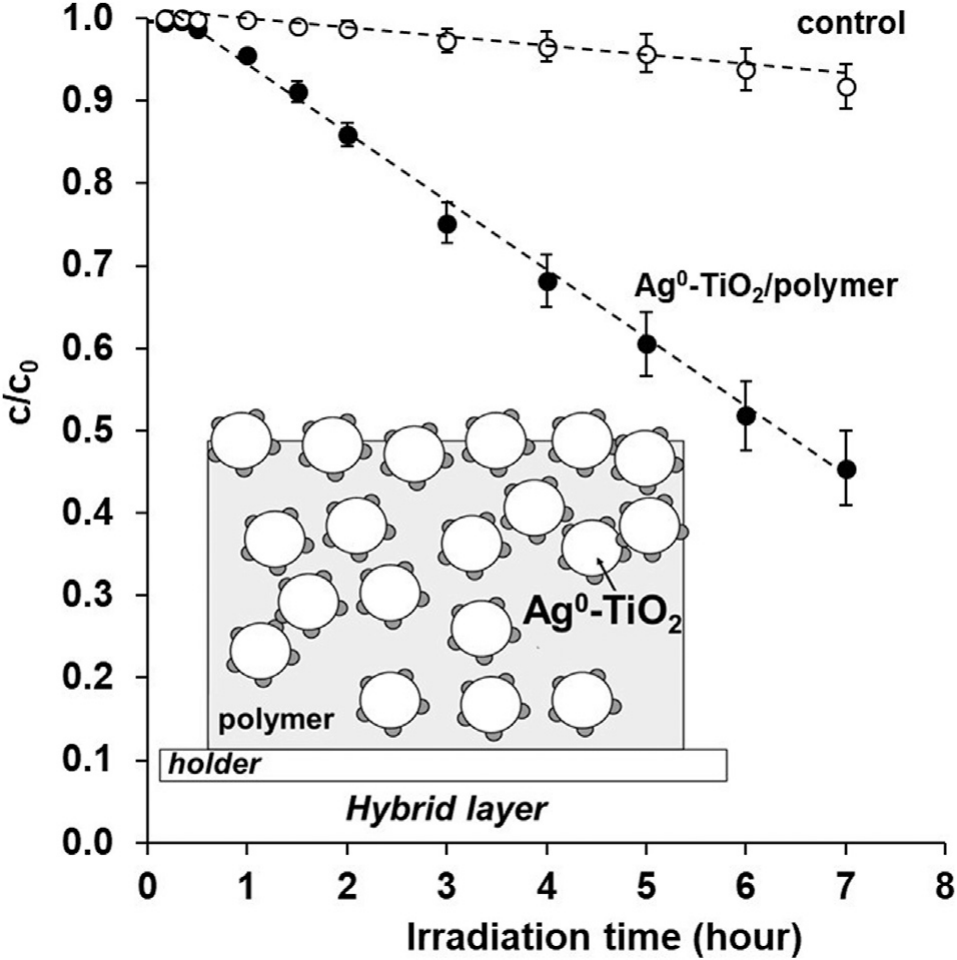
Photocatalytic degradation rate of benzoic acid test molecules on Ag^0^-TiO_2_-containing polymer-based nanohybrid thin films as a function of irradiation time under visible-light illumination. Inset: scheme representing the nanohybrid film.

In the following Figure 4, we want to prove that the irradiated photoreactive coating not only produces free radicals but it also can photocatalytically degraded simple molecules –e.g., benzoic acid as a test molecule–. The specific surface amount of the investigated nanohybrid films prepared on the 5 cm^2^ × 5 cm^2^ glass plates was 1 ± 0.2 mg cm^−2^ in all cases and the photocatalyst/polymer ratio was 60:40 wt%. The measured thickness of the photoreactive composite film was 1.45 ± 0.1 μm, while in the case of the pure polymer it was only 1.02 ± 0.2 μm. According to the results it can be stated that after an irradiation time of 7 h, approx. 55–60% of the initial benzoic acid (c_0_ = 20 ppm) was photodegraded in aqueous solution, i.e., the light induced surface free radicals can photocatalytically decompose the small molecules adsorbed on the surface of photoreactive coatings.

**FIGURE 5:**
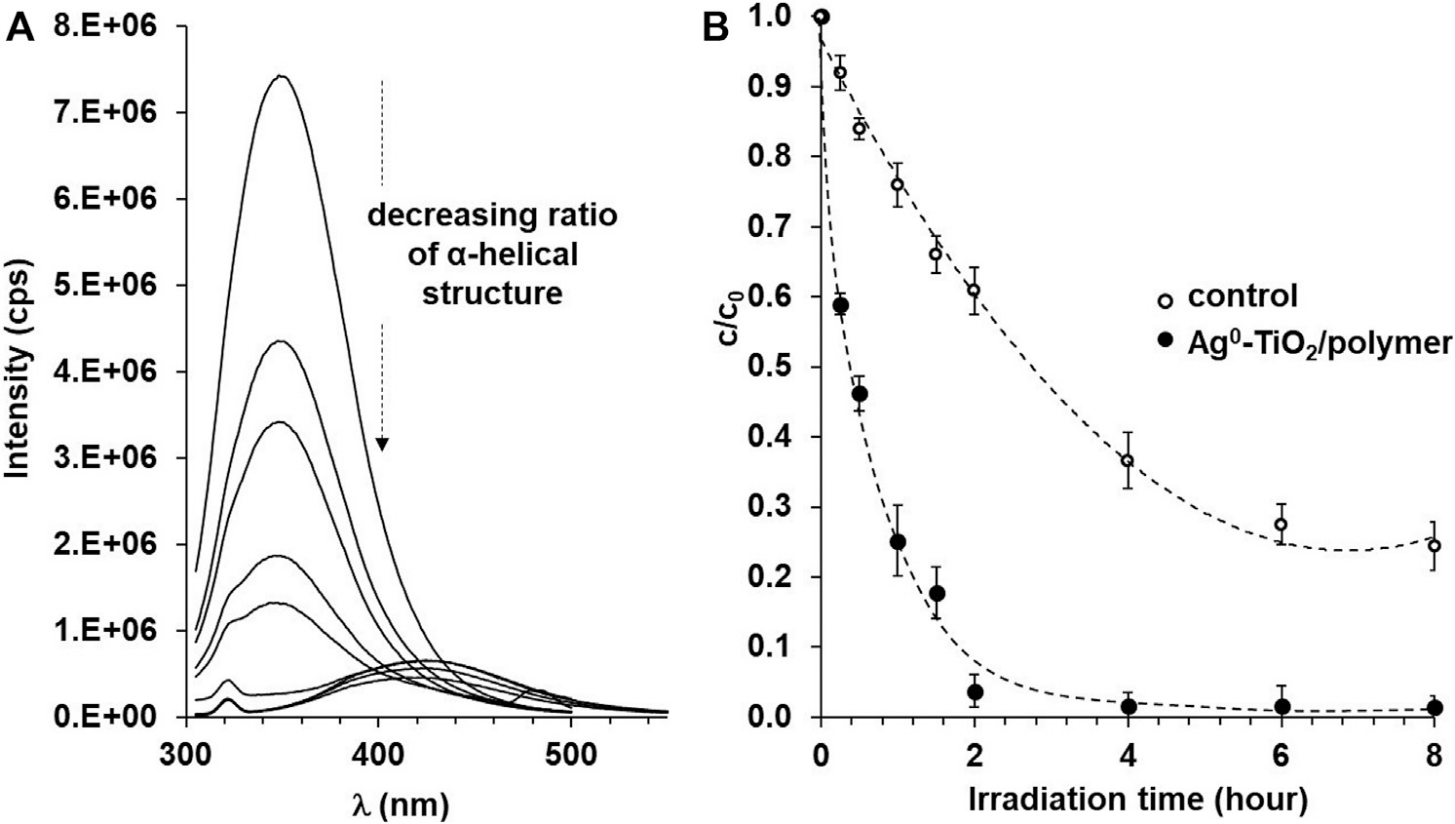
The decreasing fluorescence spectra of the BSA test protein **(A)** and the photocatalytic degradation rate of BSA **(B)** on Ag^0^-TiO_2_/polymer nanohybrid thin films as a function of irradiation time under visible-light illumination.

Next the photocatalytic efficiency of the prepared composite layer was also proved in the case of a model- biomacromolecule (BSA) with significantly higher molecular weight (M_w_ = 66 kDa), as well. Figure 5 shows the concentration decrease of 100 ppm aqueous BSA protein solution under 8 h illumination irradiated with the light source shown in Figure 1C. It can be seen that the concentration of the BSA solution without catalyst also decreases upon irradiation on the surface of the polymer composite film. If the BSA protein solution is examined under exposure, the rate of degradation increases significantly and decreases to 3.8% of the original concentration after 2 h of exposure, while after 6 h of irradiation, practically nothing can be detected from the original protein in the reaction mixture. This high photooxidation efficiency of the studied BSA protein is not only due to the improved photoreactivity of the Ag^0^-TiO_2_ plasmonic photocatalyst (Figure 1B) but certainly caused by the enhanced surface adsorption of polymer chains on the Ag^0^-TiO_2_ particles. This interaction between nanoparticles and biomacromolecules was also investigated and described by Mariam et al. (2011). In this study silver nanoparticles and BSA interaction was examined at physiological pH in an aqueous solution using fluorescence spectroscopy. They presented that the silver nanoparticles have a strong ability to quench the intrinsic fluorescence of BSA by both static and dynamic quenching mechanisms which is due to the formation of a complex between BSA and silver nanoparticles. Thus the role of surface AgNPs on the titania is twofold: on the one hand increase the photocatalytic efficiency (Figure 1B), in addition to enhance the interaction between the macromolecules and Ag^0−^TiO_2_ particles trough the above mentioned complex formation. All this together results in a strong protein photodegrading effect. Given that we have previously found that protein molecules are photocatalytically degradable and that concentrations calculated from intensities measurable in the characteristic UV spectrum decrease, it is assumed that free radicals formed in a protein solution cause a change in protein structure that also alters its secondary structure.

CD spectroscopy experiments gives information about conformational changes in BSA. The two negative bands at 208 nm (π–π* transition) and 222 nm (n-π* transition) in CD spectrum of free BSA are characteristic of the protein α-helical structure (Yu et al., 2019), whose content can be estimated by:

$$\alpha -\text{helix}\;\left(\%\right)=\frac{-MRE_{208}-4000}{33000-4000}\;\times 100,$$ (3)

where MRE_208_ is the MRE value observed at 208 nm and 4,000 is the MRE value of the *β* shape and random coil confirmation at 208 nm. The 33,000 is the MRE value of the pure α-helix at 208 nm.

**FIGURE 6:**
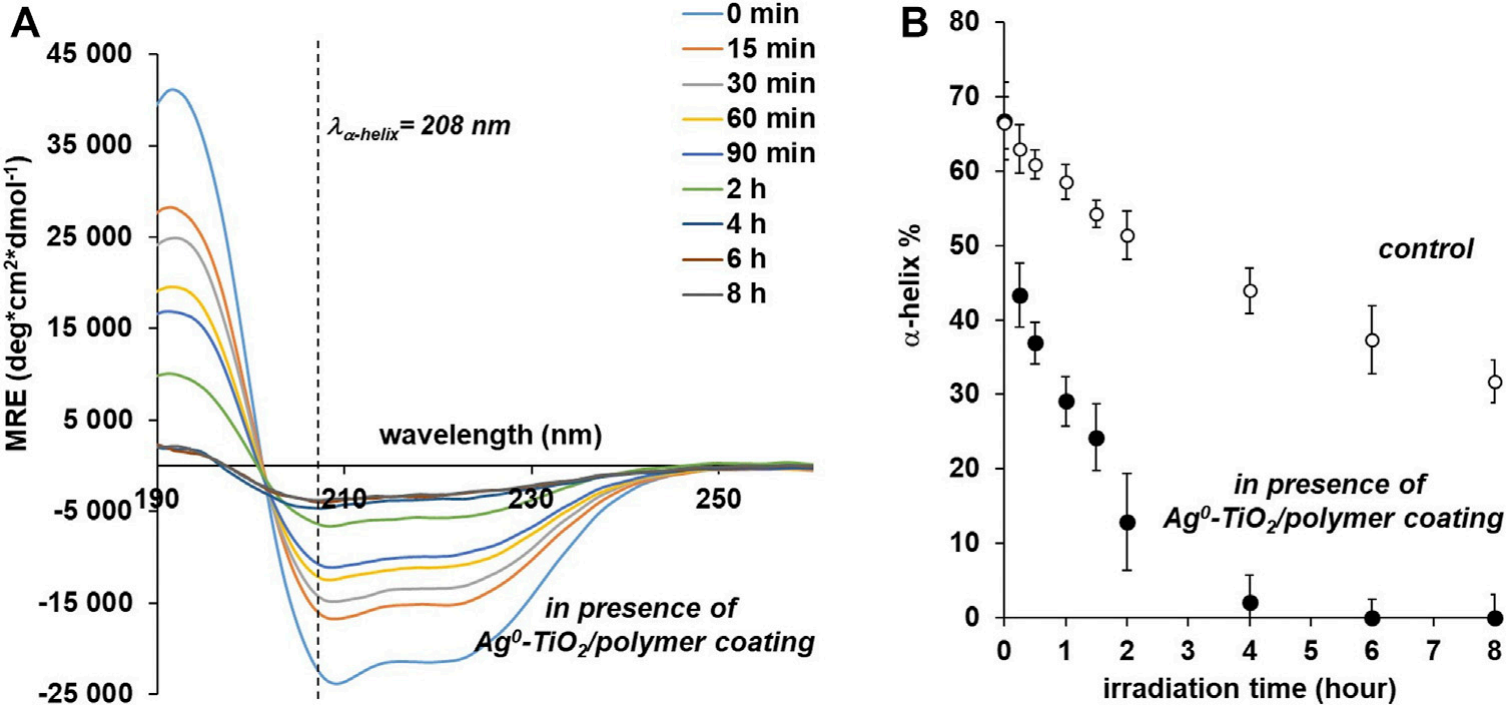
CD spectra **(A)** and the α-helix% **(B)** of BSA under photocatalytic degradation test on Ag^0^-TiO_2_/polymer nanohybrid coating as a function of irradiation time under visible-light illumination.

To prove the BSA conformational changes, CD spectra were determined in solutions from photocatalytic measurements. This gives the possibility that the percentage of α-helix in the structure of proteins changes with the transformation of the structure with the knowledge of the CD spectra. It can be seen from Figure 6 that the α-helix ratio decreases from the original 68–30% after 8 h of illumination if it does not contain the catalyst. So under the influence of light we can already expect a structural change in the structure of proteins. However, in the case of illuminated polymer composite film, the α-helix ratio decreases significantly and the α-helix structure can no longer be detected in the protein molecule after exposure for practically 4 h. This experiment was considered necessary because we wanted to point out that the structure of the proteins in the viruses examined in the next section also changes on the reactive photocatalyst composite surface, which also results in a structural change in the virus that eliminates its infectivity.

### Antiviral Effect of the Photoreactive Surface at Room Temperature

**FIGURE 7:**
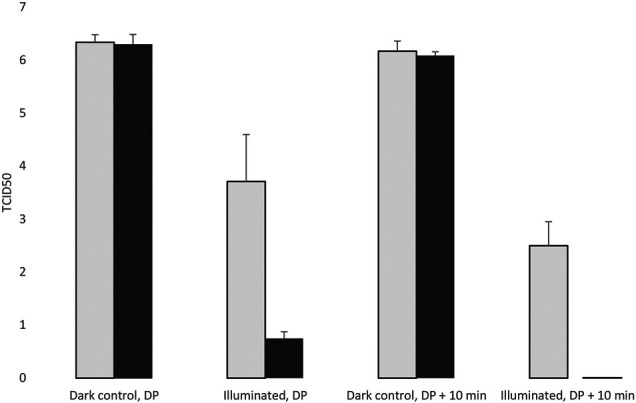
Evaluation of the virucidal effect of the photoreactive surface at room temperature. DP: Desiccation point.

In this experiment, we evaluated the effect of a photoreactive surface (Ag^0^-TiO_2_/polymer composite film) on the infectivity of pseudorabies virus (Tombácz et al., 2014), which normally infects the host organisms via airborne transmission through small particulates (aerosols and droplets). The experiments were carried out at room temperature (RT) or at 4°C before or after the desiccation of the virus suspension on the plates. Both the photoreactive and the control surfaces were either illuminated throughout the entire course of experiments or were kept in complete darkness. In the experiment carried out at RT, we observed a drastic effect of the light on the infectivity of the virus on the photoreactive surface compared to the control surface at both the liquid and the dried states of the virus inoculum. It can be seen in Figure 7 and Table 1 that light exerts a differential effect on the survival of the virus even before the drying of virus suspension. On average more than three orders of magnitude infectious virus particles were collected from the illuminated untreated surfaces than from the photoreactive surfaces. It can also be seen that light itself causes a virucidal effect even at the control surfaces. No infectious viral particles were detected 10 min after the desiccation in the photoreactive surface, whereas a significant proportion of infectious virus survived at the control surface. The light also exerts an antiviral effect at the dried state even at the control plate. It can also be seen that the desiccation itself does not have a virucidal effect during the examination period, since the viral titer was not decreased substantially in dark condition in any of the surfaces.

**TABLE 1:** The TCID50 values in three replicates, surfaces were incubated at room temperature.

	1	2	3	Mean	SD
Control, Illuminated, DP	4.50	2.75	3.88	3.71	0.89
Photoreactive, Illuminated, DP	0.75	0.625	0.875	0.75	0.13
Control, Illuminated, DP+10min	2.125	2.375	3	2.50	0.45
Photoreactive, Illuminated, DP+10min	0	0	0	0.00	0.00
Control, dark, DP	6.25	6.50	6.25	6.33	0.14
Photoreactive, dark, DP	6.50	6.13	6.25	6.29	0.19
Control, dark, DP+10min	6.00	6.13	6.38	6.17	0.19
Photoreactive, dark, DP+10min	6.13	6.00	6.13	6.08	0.07

DP: Desiccation point.

### Virucidal Effect of the Photoreactive Surface at 4°C

**FIGURE 8:**
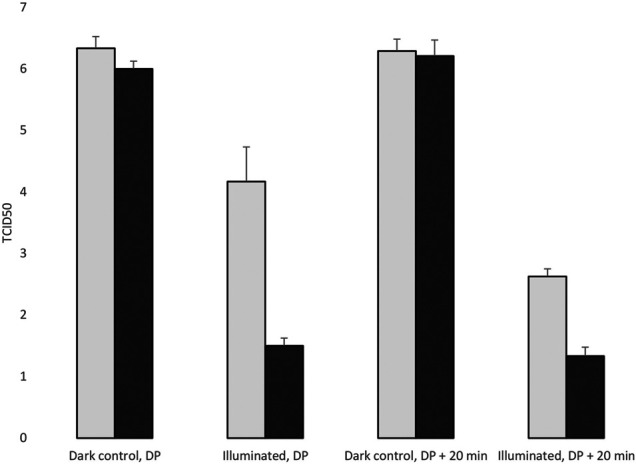
Evaluation of the virucidal effect of the photoreactive surface at 4°C. DP: Desiccation point.

At 4°C the effect of light on photoreactive surface was somewhat less than at RT before desiccation of the virus suspension. We obtained more than two orders of magnitude differential virucidal effect on the enlighted photoreactive surfaces than on the control plates (Figure 8, Table 2). The antiviral effect remained significant after drying. This experiment also demonstrate the effect of the light on the viral infectivity, because the viral titer significantly decreased in the illuminated surfaces, including the control plates. The desiccation itself did not exert a virucidal effect during the examination period.

**TABLE 2:** The TCID50 values in three replicates, surfaces were incubated at 4°C.

	1	2	3	Mean	SD
Control, Illuminated, DP	4.75	4.13	3.63	4.17	0.56
Photoreactive, Illuminated, DP	1.625	1.5	1.375	1.50	0.13
Control, Illuminated, DP+20min	2.5	2.75	2.625	2.63	0.13
Photoreactive, Illuminated, DP+20min	1.25	1.5	1.25	1.33	0.14
Control, dark, DP	6.38	6.50	6.13	6.33	0.19
Photoreactive, dark, DP	6.12	6.25	6.50	6.29	0.19
Control, dark, DP+20min	6.00	6.13	5.88	6.00	0.13
Photoreactive, dark, DP+20min	6.50	6.13	6.00	6.21	0.26

DP: Desiccation point.

### Structural Properties and Mechanical Durability of the Photoreactive Coating

**FIGURE 9:**
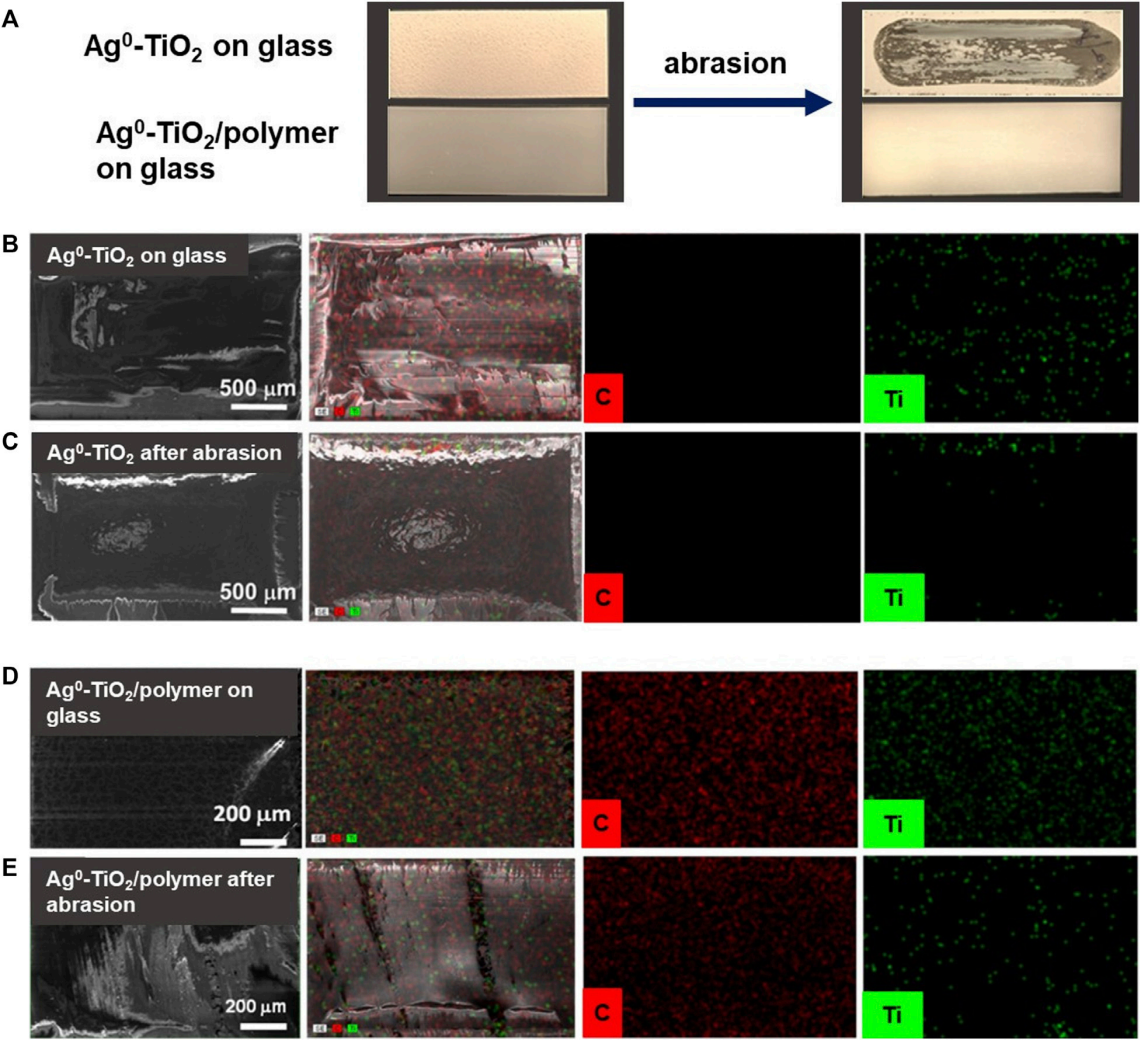
The photos **(A)** and the corresponding SEM and EDX images for carbon (red) and titania (green) content of the pure Ag^0^-TiO_2_ **(B, C)** and polyacrylate based composite (60 wt% Ag^0^-TiO_2_/40% polymer) **(D, E)** films before and after the abrasion test.

**FIGURE 10:**
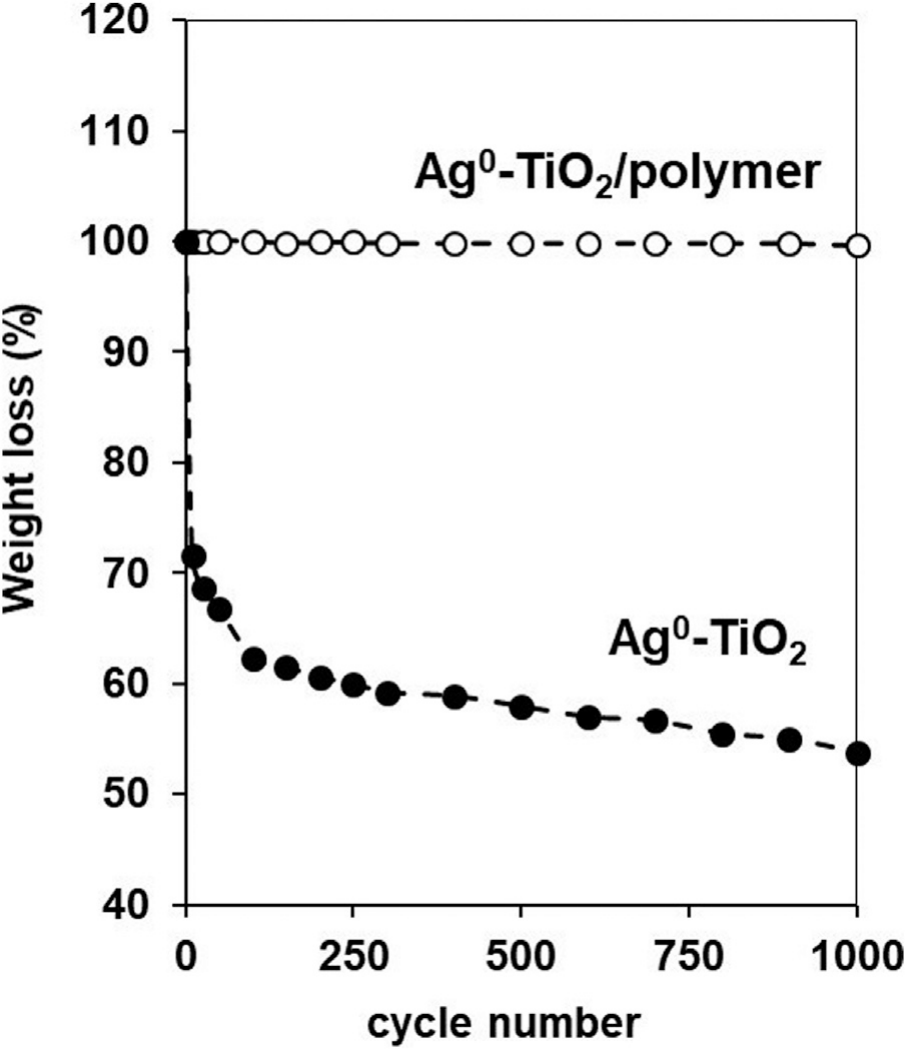
The measured weight-loss values of Ag^0^-TiO_2_ and Ag^0^-TiO_2_/polyacrylate (=60:40 wt%) coatings as a function of abrasion cycle applied on layers.

Beside the above reported antiviral properties, the structural and mechanical behaviors of the composite layer should also be considered for the more complete characterization of the films. It was reported earlier in our previous papers that at this composition (60% Ag^0^-TiO_2_/40% polymer) both the carbon of the polyacrylate and the Ti content of the photocatalyst expressed on the surface (Mérai et al., 2019). Here we demonstrated that this dual presence of the components at optimal composition resulted surfaces with simultaneous photocatalytic and mechanically durable properties. Figure 9 shows the photos and the corresponding SEM images as well the elemental mapping for carbon (red) and titania (green) content of the composite surface with 60 wt% Ag^0^-TiO_2_ content. The pure Ag^0^-TiO_2_ (without polymer) was also presented for reference. As it can be seen the before the abrasion test both the pure Ag^0^-TiO_2_ and the Ag^0^-TiO_2_/polyacrylate films were exhibited the evenly and continuous distribution of the photocatalyst particles and the polymer. However, the vulnerability of the pure Ag^0^-TiO_2_ layer is also clearly visible on the photo since after the abrasion test the layer was completely destroyed. According to the percentage weight-loss measurement (Figure 10) the layer mass was decreased very sharply, especially during the first few abrasion cycles. On contrast, if we applied polymer for the facilitation of photocatalyst particles immobilization, the mass loss of the composite film was negligible (Figure 10) and the Ag^0^-TiO_2_ particles (and the polymer) were completely covered the surface (Figure 9) even after 1,000 abrasion cycles. Thus, it can be conclude that the photoreactive layer presented here shows not only obvious antiviral properties but its mechanical durability also enables the potential practical use of the coating.

Furthermore it should also be emphasized that even the presence of the organic polymer content, the composite layers show long- term photocatalytic activity. In our previous paper we studied the reusability of the photoreactive thin films at S/L interface by measuring the discoloration of artificially created dye stains (Mérai et al., 2019). The results indicated that the photodegraded amount of dye was not reduced significantly after even 5 cycles.

## Discussion

From the result, it can be concluded that the synthetized plasmonic Ag^0^-TiO_2_ photocatalyst containing composite layers are able to produce enough surface ROS (∼80 mM/m^2^ H_2_O_2_ equivalent) for the efficient photooxidation of small (e.g benzoic acid)- or macromolecules (e.g., BSA), even under very low (<25 W/m^2^) light intensities. Thus, it was presented that the surface immobilized Ag^0^-TiO_2_ photocatalyst particles with visible light activity create the opportunity for the preparation of effective and durable antiviral surfaces. The light- induced photocatalyst particles have a virucidal effect of which extent is dependent on the virus species and family. At the epidemic seasons the light intensity is typically low, therefore the viruses retain their infectivity for prolonged periods. In this study, we demonstrated that illuminated photoreactive surfaces exerts a significant antiviral effect in both liquid and dried states using an alphaherpesvirus as a model organism for the experiment. Utilization of photoreactive surfaces can prevent infection by those viruses, which is able to spread in a contact-dependent manner.

## Data Availability

The raw data supporting the conclusions of this article will be made available by the authors, without undue reservation.
